# The Prevalence, Correlates, Detection and Control of Diabetes among Older People in Low and Middle Income Countries. A 10/66 Dementia Research Group Population-Based Survey

**DOI:** 10.1371/journal.pone.0149616

**Published:** 2016-02-25

**Authors:** Aquiles Salas, Daisy Acosta, Cleusa P. Ferri, Mariella Guerra, Yueqin Huang, K. S. Jacob, Ivonne Z. Jimenez-Velazquez, Juan J. Llibre Rodriguez, Ana L. Sosa, Richard Uwakwe, Joseph D. Williams, A. T. Jotheeswaran, Zhaorui Liu, A. M. Lopez Medina, Rosa Maria Salinas-Contreras, Martin J. Prince

**Affiliations:** 1 Medicine Department, Caracas University Hospital, Faculty of Medicine, Universidad Central de Venezuela, Caracas, Venezuela; 2 Universidad Nacional Pedro Henriquez Ureña (UNPHU), Internal Medicine Department, Geriatric Section, Santo Domingo, Dominican Republic; 3 Universidade Fedral de São Paulo, Department of Psychobiology, Sao Paulo, Brasil; 4 Psychogeriatric Unit, National Institute of Mental Health “Honorio Delgado Hideyo Noguchi”, Lima, Peru; 5 Peking University, Institute of Mental Health, Beijing, China; 6 Christian Medical College, Vellore, India; 7 Internal Medicine Dept., Geriatrics Program, School of Medicine, Medical Sciences Campus, University of Puerto Rico, San Juan, Puerto Rico; 8 Facultad de Medicina Finlay-Albarran, Medical University of Havana, Havana, Cuba; 9 National Institute of Neurology and Neurosurgery of Mexico, National Autonomous University of Mexico, Mexico City, Mexico; 10 Nnamdi Azikiwe University Teaching Hospital, Nnewi, Anambra State, Nigeria; 11 Department of Community Health, Voluntary Health Services, Chennai, India; 12 Public Health Foundation of India, New Delhi, India; 13 Policlinico 19 de abril, Plaza, La Habana, Cuba; 14 King’s College London, Institute of Psychiatry, Health Service and Population Research Department, London, United Kingdom; Cardiff University, UNITED KINGDOM

## Abstract

**Background:**

Little is known of the epidemiology of diabetes among older people in low and middle income countries. We aimed to study and compare prevalence, social patterning, correlates, detection, treatment and control of diabetes among older people in Latin America, India, China and Nigeria.

**Methods:**

Cross-sectional surveys in 13 catchment area sites in nine countries. Diagnosed diabetes was assessed in all sites through self-reported diagnosis. Undiagnosed diabetes was assessed in seven Latin American sites through fasting blood samples (glucose > = 7mmol/L).

**Results:**

Total diabetes prevalence in catchment sites in Cuba (prevalence 24.2%, SMR 116), Puerto Rico (43.4%, 197), and urban (27.0%, 125), and rural Mexico (23.7%, 111) already exceeds that in the USA, while that in Venezuela (20.9%, 100) is similar. Diagnosed diabetes prevalence varied very widely, between low prevalences in sites in rural China (0.9%), rural India (6.6%) and Nigeria (6.0%). and 32.1% in Puerto Rico, explained mainly by access to health services. Treatment coverage varied substantially between sites. Diabetes control (40 to 61% of those diagnosed) was modest in the Latin American sites where this was studied. Diabetes was independently associated with less education, but more assets. Hypertension, central obesity and hypertriglyceridaemia, but not hypercholesterolaemia were consistently associated with total diabetes.

**Conclusions:**

Diabetes prevalence is already high in most sites. Identifying undiagnosed cases is essential to quantify population burden, particularly in least developed settings where diagnosis is uncommon. Metabolic risk factors and associated lifestyles may play an important part in aetiology, but this requires confirmation with longitudinal data. Given the high prevalence among older people, more population research is indicated to quantify the impact of diabetes, and to monitor the effect of prevention and health system strengthening on prevalence, treatment and control.

## Introduction

In the USA National Health and Nutrition Examination Survey (US NHANES) 1999–2002 [1], the prevalence of total (diagnosed and undiagnosed) diabetes increases sharply with age, from 2.4% in those aged 20–39 years to 21.6% among those aged 65 years and over. Prevalence of total diabetes had increased from 5.1% (1988–1994) to 6.5% (1999–2002), with the largest increases occurring in the oldest age groups [1]. The proportion of total diabetes that was diagnosed, 70%, did not vary significantly with age.

There are few epidemiological studies of diabetes among older people in low and middle income countries (LMIC). Nationally representative surveys in China [2] and Mexico (Encuesta Nacional de Salud 2000—ENSA[3,4]) provide age-stratified estimates for older adults. In China total diabetes prevalence rose from 3.2% (20–39 years) to 20.4% for those aged 60 or over [2]. Prevalence was lower in the least economically developed rural settings. In Mexico, total diabetes prevalence was 1% at 20–29 years rising to 23% at ages 60–79 [3]. From these prevalences it was estimated that 933 thousand (40%) of the 2.3 million people with diabetes in Mexico were aged 60 and over [4]. In the Salud Bienestar y Envejecimiento (SABE) study prevalence of diagnosed diabetes was assessed by self-report, in representative samples of people aged 60 and over in seven Latin American and Caribbean cities [5]. Prevalence varied between 12.2% and 21.6%, higher in Bridgetown, Mexico City and São Paulo than in Havana, Buenos Aires, Santiago, and Montevideo.

In both the Mexican ENSA [3], and SABE Latin American surveys [5] diabetes prevalence peaked after the age of 60 and then declined among the older old (after 80 years in Mexico, and after 70 years for the SABE surveys), a pattern not apparent in China [2]. Prevalence was higher among women for all age groups in the Mexican ENSA [4], while in most SABE sites there was no sex difference [5]. In China, prevalence was significantly higher among men than women in younger age groups, but among those aged 60 and over the trend was slightly in the reverse direction [2]. In Mexico and China total diabetes prevalence was higher among the least educated, although this association was not age-stratified. In SABE prevalence of diagnosed diabetes was higher among the least educated in Buenos Aires and Mexico City, with strong trends in this direction for all sites other than Sao Paulo and Santiago [5]. In the Chinese survey total diabetes was cross-sectionally associated with overweight and obesity, systolic blood pressure and higher serum triglyceride—none of these associations was reported stratified by age. In SABE there was a consistent association between overweight (BMI 25.0–29.9) but not obesity (BMI > = 30.0) and diagnosed diabetes [5].

For China, no age-stratified data were provided on detection and control [2]. In the sample as a whole only 31% of cases were diagnosed; of these, 81% were using insulin or oral hypoglycaemic agents and 15% lifestyle interventions alone. In Mexico, the proportion diagnosed rose with age, from approximately two-thirds of those under 50, reaching 86% of those aged 60–69, 87% of those aged 70–79, and 80% of those aged 80 and over. However, the proportion of diagnosed cases controlled was lower among older than younger participants, 58% among those aged 60–69, 45% among those aged 70–79 and 50% among those aged 80 and over [4].

There is a need for more comprehensive and up-to-date information on the epidemiology of diabetes among older people, in the context of the demographic and health transitions in LMIC. It will be important to monitor social patterning, particularly the extent to which the burden shifts towards the poor and disadvantaged. Associations with modifiable metabolic and lifestyle-related risk factors should inform prevention strategies. Finally, the control of diabetes, as with other chronic diseases, depends upon well-functioning primary healthcare services [6]–the detection of diabetes and effective treatment of diagnosed cases being important indicators of progress. In the 10/66 Dementia Research Group (10/66 DRG) population-based studies, we set out to assess these parameters in a comprehensive epidemiological evaluation of diabetes and diabetes care among older people in 13 catchment areas in six Latin American countries, India, China and Nigeria.

## Materials and Methods

We present analyses of baseline survey data on the prevalence, correlates and treatment of diagnosed diabetes from catchment area surveys of participants aged 65 years and older in urban sites in Cuba (Havana and Matanzas), Dominican Republic (Santo Domingo), Puerto Rico (Bayamon), Venezuela (Caracas), urban and rural sites in Peru (Lima and Canete), Mexico (Mexico City and Morelos state), China (Xicheng and rural Daxing) and India (Chennai and Vellore) and a rural site in Nigeria (Anambra). For convenience, these sites are referred to subsequently by their country and urban or rural location. Fasting blood samples were collected in a subset of seven Latin American sites (Cuba, Dominican Republic, Puerto Rico, Venezuela, urban Peru, and urban and rural Mexico), for which we are also able to report the prevalence of undiagnosed diabetes, and the extent of control among diagnosed cases. We used a fluoride oxalate sample bottle for glucose estimation and a clot sample for other biochemistry including lipids.

10/66 DRG population-based survey protocols are detailed elsewhere [7]. Mapping of catchment areas was carried out in each site, and after door-knocking to identify eligible persons (all residents aged 65 years and over) we carried out a comprehensive one phase survey. This comprised a clinical interview; a health, medical history and lifestyle interview; a cognitive assessment; a physical examination; and an informant interview. Surveying was carried out between 2003 and 2006, other than in Puerto Rico (2007–2009). The target sample size was 2,000 per country, with 1,000 per site for those countries with split urban and rural recruitment. Recruitment was by signed informed consent. The study participant whose photographic image appears in this manuscript has given written informed consent (as outlined in PLOS consent form) for this to be published. Studies were approved by local ethical committees in each country, and by the King’s College London Research Ethics Committee.

### Measures

Relevant 10/66 DRG population-based survey assessments [7] are:

Sociodemographic factors: Age at interview from participant and informant reports, and documented age, or an event calendar, grouped as; 65–69 years, 70–74 years, 75–79 years and 80 years and over. Education level was ascertained, and coded as; no education, did not complete primary, completed primary, secondary, or tertiary education. Wealth was assessed by enumerating household assets (motor vehicles; television; fridge or freezer; water and electricity utilities; telephone; plumbed toilet; plumbed bathroom), categorised into quarters of the distribution within each site.

Diabetes: Diagnosed diabetes was defined as a self-reported medical diagnosis of diabetes (answering ‘yes’ to the question “have you ever been told by a doctor that you have diabetes?”). Undiagnosed diabetes was defined as a fasting glucose of > 7 mmol/l, among those not reporting a previous medical diagnosis. Those with a self-reported medical diagnosis of diabetes and a fasting glucose of > 7 mmol/l were considered not controlled, while those below this threshold were considered controlled. ‘Total diabetes’ comprised those with diagnosed or undiagnosed diabetes. Those with diagnosed diabetes were asked “Do you need a special diet, take tablets, or have insulin injections?” and responses were coded as diet alone, oral hypoglycaemics, insulin, or no treatment. Those using both insulin and oral hypoglycaemics were coded as insulin users.

Obesity: Waist circumference was measured in centimetres using a flexible tape measure. Central obesity was defined [8] as a waist circumference of more than 40 inches (101.6 centimetres) in men and of more than 35 inches (88.9 centimetres) in women.

Dyslipidaemia: Hypertriglyceridaemia was defined as fasting triglycerides > = 150 mg/dL. For cholesterol we used HDL cholesterol: Men < 40 mg/dL, Women < 50 mg/dL. However, where cholesterol subfractions were not analysed (Venezuela, urban Peru and Dominican Republic), we used total cholesterol > = 5.2 mmol/l.

Hypertension: Those with self-reported hypertension (“have you ever been told by a doctor that you have high blood pressure?”) and/ or a blood pressure measurement meeting WHO/ International Society of Hypertension criteria (systolic blood pressure > = 140 mmHg and/ or diastolic blood pressure > = 90 mmHg) were considered to have hypertension.

### Analyses

1. We report the prevalence of diagnosed and undiagnosed diabetes by age and sex with robust standard errors and 95% confidence intervals, accounting for household clustering. We compare the prevalences of total diabetes, and diagnosed diabetes with the sex-specific prevalences for those aged 65 years and over from US NHANES [1] using indirect standardisation to generate standardised morbidity ratios (SMR) with 95% confidence intervals.
2. For sites where fasting blood samples were collected, we describe, for each site, associations between total diabetes and age (per five year band), sex (male versus female), education (per level), assets (per quarter), central obesity, hypertension, hypertriglyceridaemia and hypercholesterolaemia, using Poisson regression models to generate mutually adjusted prevalence ratios, accounting for household clustering. For other sites, we describe associations between diagnosed diabetes and age, sex, education, assets, obesity and hypertension.
3. For sites with fasting blood samples, we report the proportion of all those with diabetes who were diagnosed, the proportion of diagnosed cases that were controlled, and the proportion of all cases that were controlled.
4. For those with diagnosed diabetes, we describe, for each site, the proportion reporting treatment with diet alone, oral hypoglycaemic drugs and insulin.
5. In a gamma-distributed random effects Poisson regression, we estimated the random effect of site on the prevalence of diagnosed diabetes, and whether this was accounted for by a) sociodemographic factors (age, sex, education and assets), b) metabolic risk factors (hypertension, obesity and dyslipidaemia), or, at site level, by c) the proportion of participants using healthcare services in the last three months. A base model was extended to include sociodemographic factors, then metabolic risk factors and finally the proportion of participants using healthcare services. The heterogeneity parameter, alpha, was noted at each stage, as an indicator of the extent of random variation in prevalence accounted for by parameters other than those included in the model.

The study protocol and the consent procedures were approved by the King's College London research ethics committee and in all countries where the research was carried out: 1- Medical Ethics Committee of Peking University the Sixth Hospital (Institute of Mental Health, China); 2- the Memory Institute and Related Disorders (IMEDER) Ethics Committee (Peru); 3- Finlay Albarran Medical Faculty of Havana Medical University Ethical Committee (Cuba); 4- Hospital Universitario de Caracas Ethics Committee (Venezuela); 5- Ethics Committee of Nnamdi Azikiwe University Teaching Hospital (Nigeria); 6- Consejo Nacional de Bioética y Salud (CONABIOS, Dominican Republic); 7- Christian Medical College (Vellore) Research Ethics Committee (India); 8- Instituto Nacional de Neurología y Neurocirugía Ethics Committee (Mexico); 9- University of Puerto Rico, Medical Sciences Campus Institutional Review Board (IRB)

## Results

### Sample Characteristics

In all, 17,945 participants were included from 13 catchment area sites in nine countries. Response proportions varied between 72% and 98%. Of those responding, 17,798 (99.2%) provided information on self-reported diabetes diagnosis. We sought fasting blood samples from 12,296 participants in seven sites. The numbers and proportions providing samples analyzable for plasma glucose were as follows: Cuba (2296, 78.2%), Dominican Republic (1448, 72.0%), Puerto Rico (1569, 78.1%), Venezuela (1189, 60.5%), urban Peru (755, 54.7%), urban Mexico (817, 81.5%), rural Mexico (890, 89.0%). Characteristics of those providing, and not providing blood samples did not differ substantially, other than in the urban Peru site where the more affluent and better educated, but also those with more physical impairments were more likely to give blood samples, and in Puerto Rico where those with dementia, stroke or significant disability were less likely to give samples (S1 table). Self-reports of diagnosed diabetes were not associated with giving blood samples in any site. Demographic and health characteristics of survey participants are summarized in Table 1. Mean age varied between 71.3 and 75.1 years by site, demographic ageing being more advanced in the Latin American sites and in urban China, compared with sites in rural China and India. Women predominated over men in all sites, accounting for between 53% and 67% of participants. Educational levels were highest in sites in urban Peru (only 9% not completing primary education), Puerto Rico (23%), Cuba (25%) and Venezuela (31%). In sites in Dominican Republic, rural Mexico, rural China, rural and urban India and Nigeria more than two-thirds had not reached this level. Central obesity was more prevalent in urban than rural sites, varying between 36.4% and 57.3% in urban Latin America and China; prevalences in rural China (15.8%), urban (17.6%) and rural India (9.6%) were considerably lower. The prevalence of hypertension ranged between 52.6% (Peru) and 79.8% (Puerto Rico) in urban sites, and between 42.6% (Peru) and 56.9% (China) in rural sites. In sites in which fasting blood samples were obtained, around one-half of participants met criteria for dyslipidaemia. The proportion using community health services in the last three months varied between 6.1% (rural China) and 81.9% (Puerto Rico), with comparatively low levels of service use also seen in sites in rural Peru (28.1%), Nigeria (30.4%) and urban China (38.6%).

**Table 1:** Participants’ sociodemographic, socioeconomic and health circumstances.

Variable	Cuba	Dominican Republic	Puerto Rico	Peru urban	Peru Rural	Venezuela	Mexico urban	Mexico rural	China urban	China rural	India urban	India rural	Nigeria
**Response rate** (%)	94	95	93	80	88	80	84	86	74	96	72	98	98
**Achieved sample** (n)	2944	2011	2009	1381	552	1965	1003	1000	1160	1002	1005	999	914
**Age** (MV ^a^)	7	0	0	1	0	4	1	0	0	0	4	0	0
Mean (SD)	75.1 (7.0)	75.3 (7.5)	76.3 (7.4)	75.0 (7.4)	74.2 (7.3)	72.3 (6.9)	74.5 (6.6)	74.1(6.7)	73.9 (6.2)	72.4 (6.0)	71.3 (6.1)	72.6 (5.8)	72.7 (7.6)
**Gender** (MV)	0	2	6	0	0	33	0	0	0	0	15	0	0
Female (%)	1913 (65.0)	1325 (65.9)	1347 (67.3)	888 (64.3)	295 (53.4)	1226 (63.5)	666 (66.4)	602 (60.2)	661 (57.0)	556 (55.5)	571 (57.7)	545 (54.6)	539 (59.0)
**Education** (MV)	8	19	9	8	8	40	2	0	0	0	2	0	6
Did not complete primary (%)	730 (24.9)	1414 (71.0)	461 (23.1)	127 (9.3)	225 (41.3)	601 (31.2)	581 (58.1)	837 (83.7)	385 (33.2)	693 (69.2)	662 (66.0)	855 (85.6)	678 (74.7)
**Number of assets** (MV)	8	5	0	0	0	0	0	0	1	0	4	0	n/a ^C^
Median (interquartile range)	6 (5–6)	5 (4–6)	7 (6–7)	6 (6–6)	5 (4–6)	6 (6–7)	6 (6–7)	4 (3–6)	5 (5–6)	6 (5–7)	4 (3–5)	3 (2–4)	n/a
**Service use (MV)**	6	2	6	1	1	33	0	0	0	0	1	0	20
Used one or more community health services in past 3 months (%)	1465 (49.9)	960 (47.8)	1639 (81.9)	670 (48.6)	155 (28.1)	1211 (62.7)	721 (71.9)	646 (64.6)	448 (38.6)	61 (6.1)	566 (56.4)	677 (67.8)	272 (30.4)
**Obesity (MV)**	23	25	404	17	2	447	11	12	11	0	15	43	n/a
Central obesity (%)	1063 (36.4)	907 (45.7)	871 (54.3)	602 (44.1)	214 (38.9)	714 (47.0)	568 (57.3)	406 (41.1)	530 (46.1)	158 (15.8)	174 (17.6)	92 (9.6)	n/a
**Hypertension (MV)**	4	11	128	3	1	176	0	2	5	0	4	22	n/a
Hypertensive, or treated	2173 (73.9)	1537 (76.9)	1501 (79.8)	725 (52.6)	235 (42.6)	1423 (79.5)	694 (69.2)	565 (56.6)	734 (63.5)	570 (56.9)	687 (68.6)	445 (45.6)	n/a
**Dyslipidemia (MV)**	612	558	453	619	n/a	690	186	107	n/a	n/a	n/a	n/a	n/a
Triglyceride	758 (32.7)	282 (19.4)	512 (32.9)	n/a	n/a	554 (43.5)	408 (49.9)	417 (46.7)	n/a	n/a	n/a	n/a	n/a
Cholesterol	1275 (55.1)	597^b^ (41.1)	740 (47.6)	364 ^b^ (47.8)	n/a	776 ^b^ (60.8)	316 (38.7)	443 (49.6)	n/a	n/a	n/a	n/a	n/a

^a^. MV = missing values

^b^. HDL cholesterol subfraction was not assayed in these sites, so dyslipidaemia is based instead on total cholesterol > = 5.2mmol/l

^c^. n/a = data not available in these sites

### The Prevalence of Diabetes

**Fig 1:**
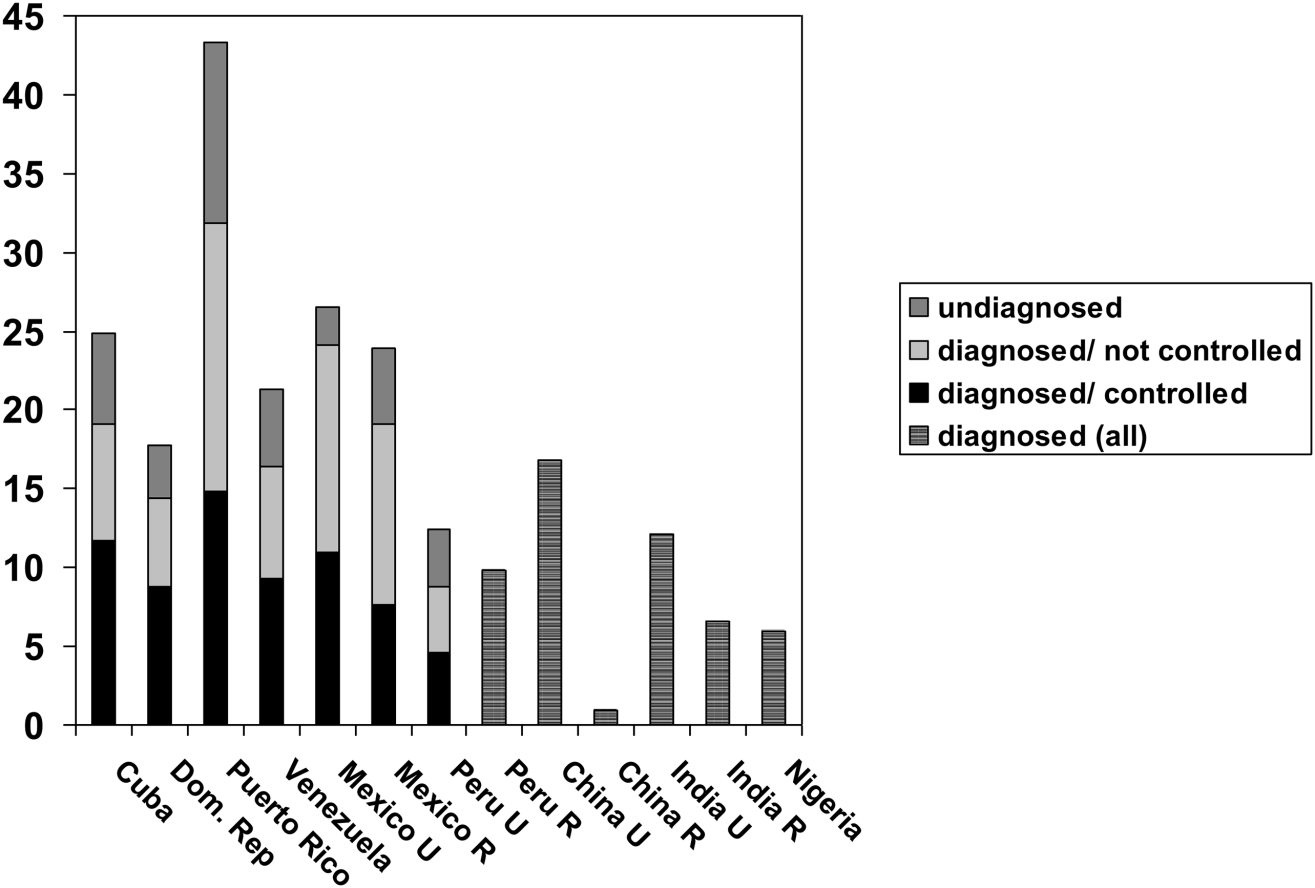
Diabetes prevalence. The prevalence of diabetes, by site according to awareness (diagnosis status) and control.

The prevalence of diagnosed diabetes (Table 2) varied very widely among sites, between 0.9% (rural China) and 32.1% (Puerto Rico). Other than rural China, a particularly low prevalence was also seen in sites in rural India (6.6%) and Nigeria (6.0%). Prevalence in Peru (8.7% in urban and 9.8% in rural) was lower than in other Latin American sites (16.0 to 24.6%). Prevalences in urban China (16.8%) and urban India (12.1%) were similar to those in Latin American sites. After standardizing for sex, compared with the prevalence recorded in US NHANES, those in sites in Cuba (117), urban Mexico (154), rural Mexico (119) and Puerto Rico (202) were higher than the USA, those in sites in Dominican Republic (88), urban Peru (55), rural Peru (62), rural China (6), urban India (76), rural India (42) and Nigeria (38) were lower, and those in sites in Venezuela and urban China were similar. For the Latin American sites where fasting blood samples were obtained, the prevalence of undiagnosed diabetes (Table 3) ranged from 2.4% (urban Mexico) to 11.6% (Puerto Rico). SMRs indicated a lower prevalence of undiagnosed diabetes in sites in Dominican Republic (62), urban Peru (64) and urban Mexico (45) compared with US NHANES, with a similar prevalence in Cuba, Venezuela and rural Mexico, and a higher prevalence in Puerto Rico (215). In these sites, the proportion of diabetes cases that were diagnosed varied from 71 to 91% (Table 4). The site-specific prevalences of total diabetes, and the SMRs compared with US NHANES were as follows; for Cuba prevalence 24.8%, SMR 116 (95% CI 107–126), Dominican Republic 17.7%, 84 (95% CI 74–94), Puerto Rico 43.4%, 197 (95% CI 183–213), urban Peru 12.5%, 58 (95% CI 47–81), Venezuela 21.3%, 100 (95% CI 88–112), urban Mexico 26.6%, 125 (95% CI 109–142), and rural Mexico 23.9%, 111 (95% CI 97–127). The prevalence of total diabetes, stratified by diagnosis is summarised in Fig 1.

**Table 2:** The prevalence of diagnosed diabetes (% with 95% confidence intervals), by age, sex and site, with standardised prevalence and morbidity ratios.

Site (gender)	Prevalence (%) by age and gender					Crude total prevalence		Standardised total prevalence	
	65–69	70–74	75–79	80+	All ages	Sample size	Total prevalence (%)	Age and sex standardised prevalence (%)	Sex adjusted SMR (95% CI)^a^
Cuba (F)	20.4 (16.8–24.0)	26.1 (22.2–29.9)	21.3 (17.4–25.3)	18.8 (15.4–22.2)	21.7 (19.8–23.5)	N = 2944^b^, MV = 16^c^	18.5 (17.1–20.0)	18.3 (17.0–19.7)	117 (107–127)
Cuba (M)	11.4 (7.6–15.2)	15.1 (11.0–19.2)	13.6 (9.1–18.0)	10.8 (6.8–14.8)	12.8 (10.7–14.8)				
Dominican Republic (F)	15.7 (11.9–19.5)	18.3 (14.1–22.5)	12.5 (8.5–16.4)	11.8 (8.6–15.0)	14.5 (12.6–16.4)	N = 2011^b^, MV = 4^c^	14.0 (12.5–15.5)	14.0 (12.7–15.8)	88 (78–99)
Dominican Republic (M)	14.4 (9.3–19.4)	14.9 (9.9–19.9)	13.6 (7.8–19.5)	8.9 (4.6–13.2)	13.0 (10.5–15.6)				
Peru Urban (F)	8.0 (4.7–11.3)	8.6 (4.9–12.3)	8.5 (4.5–12.4)	6.6 (3.3–10.0)	7.9 (6.1–9.7)	N = 1371^b^, MV = 10^c^	8.7 (7.2–10.2)	8.7 (7.2–10.3)	55 (45–65)
Peru Urban (M)	10.8 (5.0–16.6)	11.4 (5.9–16.8)	6.6 (1.9–11.3)	10.7 (5.6–15.9)	10.0 (7.4–12.7)				
Peru Rural (F)	13.0 (6.4–19.6)	15.9 (8.0–23.7)	13.2 (4.1–22.3)	8.5 (1.4–15.6)	12.9 (9.1–16.7)	N = 551^b^, MV = 1^c^	9.8 (7.3–12.3)	10.3 (7.7–12.9)	62 (47–80)
Peru Rural (M)	8.9 (2.6–15.1)	1.7 (0.0–5.1)	10.4 (1.8–19.1)	4.2 (0.0–8.8)	6.2 (3.3–9.2)				
Venezuela (F)	14.6 (11.5–17.6)	17.8 (13.3–22.2)	18.7 (13.5–23.9)	14.2 (9.4–19.0)	16.0 (14.0–18.1)	N = 1926^b^, MV = 39^c^	16.0 (14.4–17.7)	16.2 (14.5–17.9)	101 (90–113)
Venezuela (M)	16.4 (12.3–20.5)	16.0 (10.4–21.6)	12.1 (6.4–17.8)	20.7 (12.1–29.2)	16.1 (13.3–18.8)				
Mexico Urban (F)	21.1 (15.2–26.9)	27.0 (21.1–32.9)	24.6 (17.1–32.1)	25.2 (18.1–32.3)	24.5 (21.2–27.7)	N = 1002^b^, MV = 1^c^	24.6 (21.9–27.2)	24.9 (22.1–27.7)	154 (136–175)
Mexico Urban (M)	33.3 (21.4–45.3)	21.2 (13.8–28.6)	26.6 (16.8–36.3)	21.5 (12.4–30.6)	24.7 (20.1–29.3)				
Mexico Rural (F)	27.4 (21.2–33.7)	23.5 (16.7–30.3)	20.9 (14.0–27.8)	16.4 (9.8–23.0)	22.8 (19.4–26.1)	N = 1000^b^, MV = 0^c^	18.9 (16.4–21.4)	19.2 (16.7–21.6)	119 (103–137)
Mexico Rural (M)	18.6 (11.1–26.2)	12.6 (6.2–19.0)	12.6 (5.7–19.6)	8.5 (3.2–13.8)	13.1 (9.7–16.4)				
Puerto Rico (F)	31.8 (26.5–37.0)	31.7 (26.4–36.9)	31.9 (26.7–37.0)	31.1 (26.6–35.5)	31.6 (29.0–34.1)	N = 2009^b^, MV = 7^c^	32.1 (30.0–34.1)	32.2 (30.0–34.3)	202 (187–218)
Puerto Rico (M)	31.2 (22.5–39.9)	36.8 (29.2–44.4)	30.7 (23.6–37.8)	33.3 (27.2–39.5)	33.1 (29.5–36.8)				
China Urban (F)	23.0 (17.3–28.8)	21.4 (15.7–27.1)	15.3 (9.3–21.4)	11.8 (6.0–17.6)	18.9 (15.9–21.9)	N = 1159^b^, MV = 1^c^	16.8 (14.7–19.0)	16.9 (14.7–19.1)	106 (92–121)
China Urban (M)	18.0 (10.9–25.2)	18.0 (12.1–23.9)	12.8 (6.8–18.9)	5.5 (1.2–9.8)	14.1 (11.0–17.1)				
China Rural (F)	1.6 (0.0–3.3)	No cases	0.8 (0.0–2.4)	No cases	0.7 (0.0–1.4)	N = 1002^b^, MV = 0^c^	0.9 (0.3–1.5)	0.7 (0.2–1.2)	6 (3–10)
China Rural (M)	2.1 (0.1–4.1)	No cases	1.3 (0.0–3.8)	No cases	1.1 (0.1–2.1)				
India Urban (F)	15.5 (11.0–20.1)	10.6 (6.2–15.0)	10.7 (3.7–17.7)	6.1 (0.3–11.8)	12.1 (9.4–14.8)	N = 1004^b^, MV = 1^c^	12.1 (10.0–14.1)	11.3 (9.3–13.4)	76 (63–90)
India Urban (M)	12.1 (7.3–17.0)	10.3 (5.0–15.6)	9.0 (2.1–15.8)	15.4 (5.6–25.2)	11.7 (8.6–14.8)				
India Rural (F)	6.8 (3.2–10.3)	7.1 (3.5–10.8)	7.9 (2.3–13.4)	1.5 (0.0–4.3)	6.4 (4.4–8.5)	N = 999^b^, MV = 0^c^	6.6 (5.0–8.2)	6.3 (4.8–7.8)	42 (32–53)
India Rural (M)	5.8 (1.9–9.6)	7.1 (3.1–11.2)	5.7 (0.8–10.5)	9.6 (2.8–16.3)	6.8 (4.5–9.1)				
Nigeria (F)	3.9 (1.4–6.4)	6.6 (2.5–10.6)	8.2 (0.5–15.8)	7.2 (1.8–12.7)	5.6 (3.5–7.6)	N = 914^b^, MV = 67^c^	6.0 (4.4–7.6)	6.4 (4.6–8.3)	38 (28–49)
Nigeria (M)	7.2 (2.8–11.6)	10.1 (3.0–17.3)	3.2 (0.0–7.6)	5.6 (0.8–10.3)	6.6 (4.0–9.3)				

^a^. indirectly sex standardised morbidity ratios compared with USA National Health and Nutrition Survey (USA = 100, reference)

^b^. Number of survey participants

^c^. Number of survey participants lacking data on self-reported diabetes diagnosis

**Table 3:** The prevalence of undiagnosed diabetes (% with 95% confidence intervals), by age, sex and site, with standardised prevalence and morbidity ratios.

	Prevalence (%) by age and gender					Crude total prevalence		Standardised total prevalence	
	65–69	70–74	75–79	80+	All ages	Total sample	Total prevalence (%)	Age and sex standardized prevalence (%)	Sex adjusted SMR (95% CI)^a^
Cuba (F)	6.6 (4.2–9.1)	3.7 (1.9–5.5)	5.3 (2.9–7.8)	6.0 (3.6–8.4)	5.4 (4.2–6.5)	N = 2355^b^, MV = 19^c^	5.7 (4.7–6.7)	5.7 (4.7–6.6)	103 (86–121)
Cuba (M)	7.9 (4.2–11.6)	6.4 (3.3–9.5)	4.8 (1.6–8.1)	6.1 (2.6–9.6)	6.4 (4.7–8.1)				
Dominican Republic (F)	4.2 (1.8–6.6)	2.8 (0.8–4.9)	2.5 (0.3–4.7)	4.6 (2.2–7.1)	3.6 (2.5–4.8)	N = 1483^b^, MV = 3^c^	3.4 (2.5–4.3)	3.3 (2.4–4.2)	62 (46–81)
Dominican Republic (M)	4.7 (1.0–8.4)	2.3 (0.0–4.9)	3.5 (0.0–7.4)	0.9 (0.0–2.5)	2.8 (1.3–4.4)				
Peru Urban (F)	3.2 (0.4–6.0)	5.6 (1.6–9.6)	1.0 (0.0–2.8)	1.0 (0.0–2.9)	3.1 (1.5–4.6)	N = 770^b^, MV = 8^c^	3.6 (2.2–4.9)	3.3 (2.1–4.6)	64 (43–92)
Peru Urban (M)	1.9 (0.0–5.6)	7.6 (1.8–13.4)	4.9 (0.0–10.3)	2.7 (0.0–6.3)	4.5 (2.0–7.0)				
Venezuela (F)	4.2 (2.1–6.4)	4.0 (1.1–6.9)	6.3 (2.3–10.3)	4.7 (1.0–8.4)	4.7 (3.2–6.1)	N = 1284^b^, MV = 94^c^	4.9 (3.7–6.1)	4.9 (3.6–6.2)	89 (68–114)
Venezuela (M)	6.3 (2.7–9.8)	4.8 (0.7–8.9)	3.8 (0.0–7.9)	5.5 (0.0–11.5)	5.3 (3.2–7.4)				
Mexico Urban (F)	2.6 (0.1–5.1)	1.1 (0.0–2.7)	3.8 (0.1–7.4)	2.7 (0.0–5.8)	2.4 (1.1–3.6)	N = 822^b^, MV = 1^c^	2.4 (1.3–3.6)	2.5 (1.4–3.6)	45 (28–68)
Mexico Urban (M)	4.3 (0.0–10.2)	4.1 (0.2–8.0)	1.6 (0.0–4.8)	No cases	2.6 (0.7–4.6)				
Mexico Rural (F)	4.0 (1.1–6.8)	5.3 (1.5–9.1)	4.0 (0.6–7.4)	4.8 (0.7–8.9)	4.5 (2.7–6.2)	N = 895^b^, MV = 2^c^	4.8 (3.4–6.2)	4.8 (3.4–6.2)	85 (62–113)
Mexico Rural (M)	6.7 (1.5–11.8)	4.6 (0.2–8.9)	7.7 (1.8–13.6)	3.1 (0.0–6.6)	5.4 (3.1–7.8)				
Puerto Rico (F)	7.2 (4.0–10.4)	11.6 (7.6–15.6)	10.0 (6.4–13.7)	11.8 (8.1–15.4)	10.2 (8.4–12.1)	N = 1586^b^, MV = 25^c^	11.5 (9.9–13.2)	11.6 (10.0–13.2)	215 (185–248)
Puerto Rico (M)	16.7 (9.0–24.4)	15.3 (9.2–21.4)	11.3 (5.7–16.9)	14.7 (9.2–20.3)	14.4 (11.3–17.5)				

^a^. indirectly sex standardised morbidity ratios compared with USA National Health and Nutrition Survey (USA = 100, reference)

^b^. Number of participants with fasting glucose estimations

^c^. Number of participants with fasting glucose estimations lacking data on self-reported diabetes diagnosis

**Table 4:** Diabetes awareness, and control, by site.

Site	Proportion diagnosed (among those with diabetes, that is meeting WHO criteria, and/ or aware)	Proportion of those diagnosed who were controlled	Proportion both diagnosed and controlled (among those with diabetes, that is meeting WHO criteria, and/ or aware)
Cuba	438/ 569 (77.0%)	269/438 (61.4%)	269/ 569 (47.3%)
Dominican Republic	208/ 257 (80.9%)	127/208 (61.1%)	127/ 257 (49.4%)
Peru Urban	67/ 94 (71.3%)	35/67 (52.2%)	35/94 (37.2%)
Venezuela	195/ 253 (77.1%)	111/195 (56.9%)	111/ 253 (43.9%)
Mexico Urban	197/217 (90.8%)	90/197 (45.7%)	90/ 217 (41.5%)
Mexico Rural	170/213 (79.8%)	68/170 (40.0%)	68/213 (31.9%)
Puerto Rico	493/643 (76.7%)	160/463 (34.6%)	160/643 (24.9%)

### Control and Treatment of Diabetes

**Fig 2:**
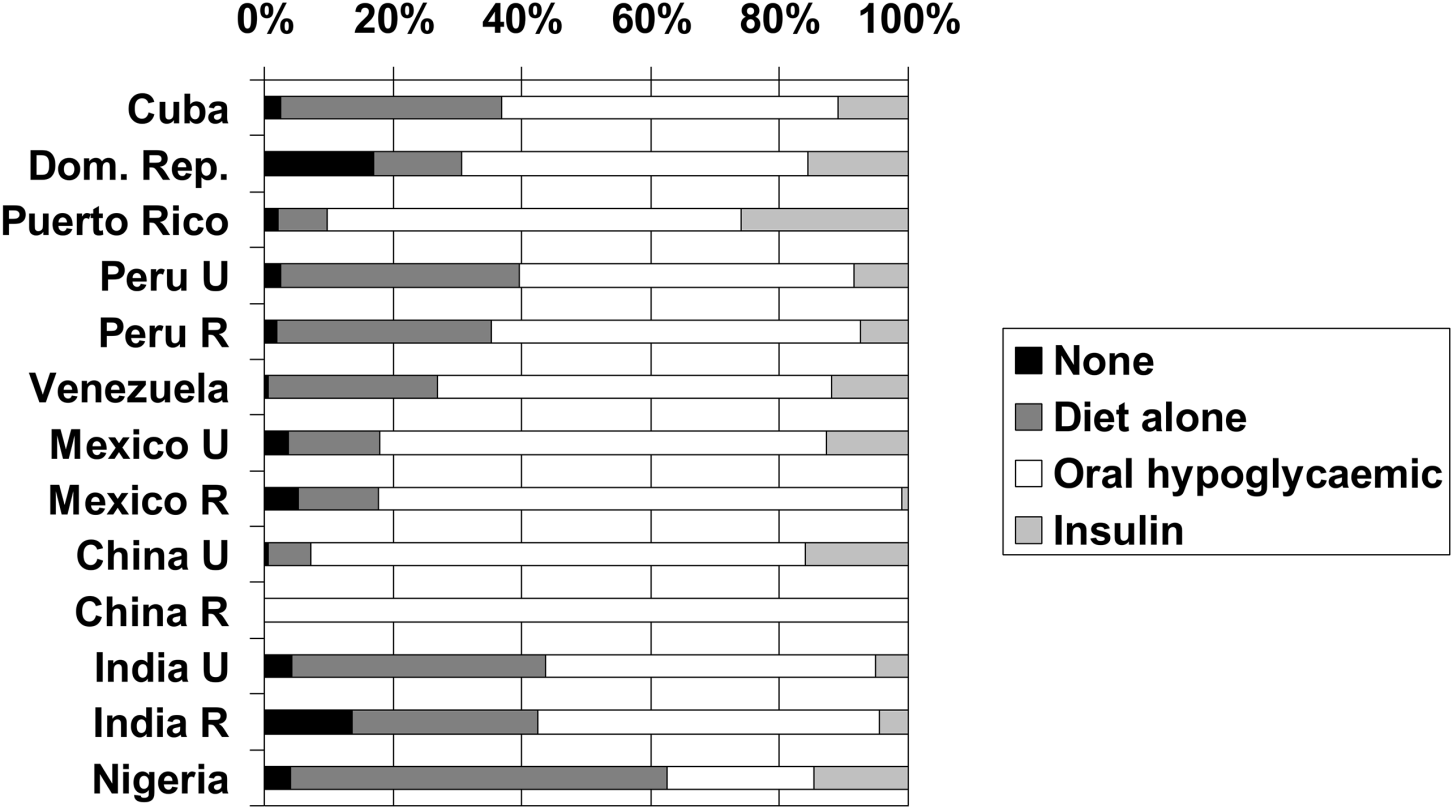
Diabetes treatment. Treatment profile of those with diagnosed diabetes, by site.

For sites with fasting blood samples, between 35% and 61% of diagnosed patients were controlled (Table 4). The proportion of all diabetes cases (diagnosed and undiagnosed) that were controlled varied from 25% to 49%. Twenty-six percent of diagnosed diabetic patients in the Puerto Rico site were prescribed insulin, compared with only 1% in rural Mexico, 5% in rural India, 6% in urban India, 7% in rural Peru and 8% in urban Peru (Fig 2). In other sites the proportion varied between 11 and 16%. In sites in Puerto Rico (90%), Mexico (82%) and urban China (93%) a particularly high proportion of diagnosed diabetic patients received pharmacological treatment with either insulin or oral hypoglycaemic agents. In the Nigeria site only 36% reported receiving pharmacological treatment.

### The Correlates of Diabetes

The prevalence of diagnosed diabetes (in sites lacking fasting plasma glucose assessments), but not total diabetes (in other sites) decreased with age (Table 5). The effect of sex was also highly heterogeneous—total diabetes was more common among women than men in Cuba and urban Mexico, while prevalence was higher among men in urban Peru and Puerto Rico. The effects of education and assets, mutually adjusted, tended in opposite directions for total and diagnosed diabetes, with a lower prevalence among the better educated, but a higher prevalence among those with more household assets. Hypertension and obesity were strongly associated with both outcomes. Hypertriglyceridaemia was strongly associated with total diabetes. The effect of hypercholesterolaemia was less evident, but there was an association with low HDL cholesterol in sites where this was measured (PR 1.18, 95% CI 1.08–1.29)

**Table 5:** Associations (prevalence ratios with 95% confidence intervals) between age, sex, education, obesity, dyslipidaemia and diabetes.

Site, and outcome	Age (per 5 year band)	Sex (Male versus Female)	Education (per level)	Household assets (per quarter)	Hypertension	Obesity	Dyslipidaemia	
							Triglyceride	Cholesterol
**Total diabetes**								
Cuba	0.96 (0.90–1.02)	0.73 (0.62–0.85)	0.94 (0.87–1.01)	1.02 (0.95–1.11)	1.22 (1.02–1.46)	1.48 (1.27–1.73)	1.40 (1.21–1.61)	1.25 ^a^ (1.07–1.46)
Dominican Republic	0.87 (0.79–0.96)	0.87 (0.68–1.17)	0.98 (0.85–1.11)	1.12 (1.00–1.26)	1.31 (0.98–1.76)	2.18 (1.68–2.81)	1.31 (1.02–1.69)	0.86 ^b^ (0.68–1.08)
Venezuela	0.98 (0.88–1.09)	1.05 (0.83–1.32)	0.90 (0.80–1.03)	1.09 (0.97–1.22)	1.29 (0.94–1.77)	1.04 (0.81–1.34)	1.78 (1.38–2.30)	0.80 ^b^ (0.62–1.02)
Mexico Urban	1.03 (1.00–1.08)	0.86 (0.78–0.95)	0.99 (0.96–1.03)	1.20 (1.10–1.32)	1.19 (0.92–1.54)	1.04 (0.81–1.33)	1.48 (1.17–1.87)	0.99 ^a^ (0.77–1.25)
Mexico Rural	0.95 (0.85–1.06)	1.08 (0.85–1.38)	0.95 (0.86–1.04)	1.01 (0.91–1.12)	1.31 (1.02–1.69)	1.33 (1.02–1.73)	1.61 (1.24–2.10)	1.23 ^a^ (0.96–1.59)
Peru Urban	0.81 (0.67–0.97)	1.55 (1.06–2.27)	0.80 (0.65–0.99)	1.14 (0.94–1.39)	1.52 (1.00–2.31)	1.77 (1.14–2.76)	n/a ^c^	0.97 ^b^ (0.66–1.44)
Puerto Rico	1.04 (0.98–1.09)	1.18 (1.03–1.34)	0.96 (0.91–1.01)	1.03 (0.96–1.11)	1.69 (1.39–2.05)	1.35 (1.18–1.54)	1.09 (0.96–1.24)	1.18 ^a^ (1.04–1.34)
Pooled estimates	1.00 (0.97–1.02)	0.94 (0.89–1.00)	0.97 (0.94–0.99)	1.07 (1.03–1.11)	1.35 (1.23–1.49)	1.38 (1.28–1.49)	1.33 (1.23–1.43)	1.09 (1.02–1.18)
Heterogeneity: Chi^2^ (df), p-value, I^2^	19.8 (6), 0.003, 69.6%	33.5 (6), <0.001, 82.1%	6.9 (6), 0.33, 12.7%	10.8 (6), 0.09, 44.8%	7.7 (6), 0.26, 22.4%	24.2 (6), <0.001, 75.2%	17.6 (5), 0.003, 71.6%	16.3 (6), 0.01, 63.1%
**Diagnosed diabetes**								
Peru rural	0.90 (0.72–1.13)	0.71 (0.31–1.66)	1.11 (0.87–1.42)	1.06 (0.84–1.35)	1.44 (0.82–2.50)	1.55 (0.69–3.49)	n/a ^c^	n/a
China urban	0.77 (0.68–0.88)	1.13 (0.83–1.54)	0.87 (0.78–0.97)	1.28 (1.06–1.56)	1.57 (1.15–2.14)	1.53 (1.14–2.06)	n/a	n/a
China rural	0.52 (0.17–1.52)	0.96 (0.22–4.25)	1.65 (0.88–3.09)	2.28 (0.97–5.33)	6.38 (0.81–50.6)	1.30 (0.23–7.25)	n/a	n/a
India urban	0.86 (0.71–1.04)	0.98 (0.67–1.42)	0.96 (0.82–1.13)	1.36 (1.12–1.65)	1.27 (0.85–1.90)	1.06 (0.67–1.65)	n/a	n/a
India rural	1.00 (0.80–1.24)	0.75 (0.43–1.30)	1.45 (1.11–1.89)	1.01 (0.79–1.29)	1.65 (0.98–2.80)	1.08 (0.48–2.43)	n/a	n/a
Nigeria	0.89 (0.53–1.51)	1.18 (0.47–2.98)	0.67 (0.28–1.62)	1.46 (1.01–2.10)	n/a	n/a	n/a	n/a
Pooled estimates	0.84 (0.77–0.92)	1.00 (0.81–1.22)	0.97 (0.89–1.05)	1.23 (1.11–1.36)	1.50 (1.22–1.84)	1.36 (1.08–1.70)	n/a	n/a
Heterogeneity: Chi^2^ (df), p-value, I^2^	5.4 (5), 0.37, 7.1%	2.4 (5), 0.79, 0.0%	17.2 (5), 0.004, 70.9%	8.1 (5), 0.15, 37.9%	2.8 (4), 0.60, 0.0%	2.2 (4), 0.70, 0.0%		
Pooled estimates (across both outcomes)	0.99 (0.96–1.01)	0.95 (0.85–1.01)	0.97 (0.95–0.99)	1.09 (1.05–1.13)	1.38 (1.27–1.50)	1.38 (1.28–1.49)		
Heterogeneity: Chi^2^ (df), p-value, I^2^	38.8 (12), <0.001, 69.1%	36.2 (12), <0.001, 66.9%	24.1 (12), 0.02, 50.1%	25.3 (12), 0.01, 52.6%	11.3 (11), 0.41, 2.7%	26.4 (11), 0.006, 58.3%		

^a^. Low HDL cholesterol

^b^. High total cholesterol (cholesterol subfractions not having been assayed in these sites)

^c^. n/a = Not assessed in these sites

### Modelling the Prevalence of Diagnosed Diabetes Across Sites

In the base model of the gamma-distributed random effects Poisson regression for the outcome of diagnosed diabetes, the random effect of site (heterogeneity parameter, alpha) was 0.45 (95% confidence interval 0.21–0.94), falling slightly to 0.41 (0.19–0.87) after adjusting for age, sex, education and assets, and then to 0.35 (0.16–0.79) after adjusting for hypertension and obesity. Accounting also for the proportion in each site using healthcare services reduced alpha to just 0.19 (0.08–0.45). For each one percent increase in the proportion using healthcare services the prevalence of diagnosed diabetes increased by a multiple of 1.026 (95% CI, 1.010–1.041). Thus, little of the variance in diagnosed diabetes prevalence between sites was explained by compositional factors, while around one-half was explained by a site-level indicator of access to healthcare.

## Discussion

### Principal Findings

The prevalence of total diabetes among older people in our catchment sites in Cuba and Mexico already exceeds that in the US NHANES, while that in Venezuela is similar. Prevalence of total diabetes was particularly high in Puerto Rico. Other than possible artefacts arising from selection of atypical catchment areas, the low prevalence of diagnosed diabetes that we observed in sites in rural China, rural and urban India and Nigeria may reflect a low prevalence of total diabetes, or low levels of detection, or both. In a previous study in Southern Nigeria, in which glucose tolerance tests were completed on those with high random glucose, the prevalence of total diabetes in those aged 50 and over was 12.8%, substantially higher than the 6.0% of those aged 65 and over reporting a diagnosis in our study [9]. In our Latin American sites in which blood tests were carried out, three-quarters or more of cases in the community had been diagnosed. However, only 40–61% of diagnosed cases were currently controlled. While in most sites other than Nigeria, a high proportion of diagnosed patients reported receiving pharmacotherapy, insulin was rarely used in sites in rural Mexico, Peru, India and rural China. The tendency for the prevalence of diabetes to be lower among the oldest old was only apparent for diagnosed diabetes, and likely reflected an ascertainment bias. When the effects of education and assets were considered together, and mutually adjusted, diabetes tended to more common among the least educated and the wealthiest. Hypertension, central obesity, hypertriglyceridaemia, low HDL cholesterol (but not hypercholesterolaemia) were consistently cross-sectionally associated with diabetes.

### Strengths and Weaknesses of the Study

The main strengths are the focus on older people, the range of countries and settings surveyed, and the comprehensive appraisal of prevalence, social patterning, risk factors, and detection and treatment, all in the same survey using standardised protocols across 13 catchment areas in nine LMIC. We have contributed particularly to the delineation of the diabetes epidemic in hispanic Latin American and Caribbean countries. Previous research had either focused on diagnosed diabetes alone [5], or in the case of the Mexican ENSA survey [3,4], provided little information specific to older people. The main weakness is the use of catchment area rather than nationally or regionally representative samples. This means that we cannot safely generalize our findings to the country as a whole in which the research was conducted. We would have liked to have obtained fasting blood samples in all sites but funding did not permit this outside of Latin America. Diagnostic precision might have been enhanced if oral glucose tolerance tests had also been carried out, but this would have been unwieldy in the community-setting of these epidemiological studies. Evaluation of diabetic control among diagnosed and treated cases was hampered by the lack of HbA1C assessments, which would have given a better summary of recent control than a one-off fasting glucose.

### Contextualisation with Other Research

The prevalence of total diabetes in our rural and urban Mexican sites was higher than that recorded nationally in ENSA, perhaps reflecting their particular characteristics, or the six years passage of time and the evolution of the health transition in that country. The prevalence of diagnosed diabetes in Havana (18.5%) was also higher than that recorded for a slightly younger sub-group in the same city in SABE four years earlier (12.5%), and the same was true for Mexico City (24.6% versus 21.0%). While the Mexican ENSA and Chinese national surveys suggested a similar prevalence among older people to that in the US NHANES, our results suggest a significantly higher prevalence among older people in Cuba, Mexico and Puerto Rico (where the prevalence of diagnosed diabetes alone exceeded that of total diabetes in any other country in the region). This is consistent with the finding from US NHANES that among older people, prevalence is higher among ‘Mexican Americans’ (32.7%), and non-Hispanic blacks (35.7%) than among non-Hispanic whites (20.3%) [1]. The effect of ethnicity on diabetes prevalence in the US was explored in more detail in the National Health Interview Survey 2000–2005 [10]. The particularly high prevalence of self-reported diabetes among Puerto Rican and Cuban-born migrants was not apparent in those of Puerto Rican and Cuban ancestry born in the USA. Culturally determined dietary and lifestyle factors may have played an important part. Others have noted a very high prevalence of metabolic syndrome in Puerto Rico, particularly among older people [11].

The persistence, into old age, of obesity as an aetiologic factor for diabetes was partly supported in a median 12-year follow-up of participants in the Cardiovascular Health Study [12]. Adiposity, and weight gain during middle-age and older age were associated with incident diabetes. However, relative risks were substantially smaller for those over 75 years compared with those aged 65–74 years at baseline. Our findings support the salience of central obesity, hypertension and dyslipidaemia to diabetes among older people in LMIC, although longitudinal studies would be required to establish direction of causality. The cross-sectional clustering of these metabolic disorders supports the applicability of the concept of metabolic syndrome to older people in LMIC. However, recent critiques highlight the need to identify underlying mechanisms, clarify definitions and cut-points, and establish whether metabolic syndrome provides better overall risk stratification than its individual components [13]

### Future Research

Our analysis highlights the need for more research. Our international perspective has shown that the prevalence of diagnosed diabetes is strongly influenced by the extent of use of health services prevailing in that site. Limited help-seeking and/ or access to services, and poor recall of diagnoses will compromise diagnosed diabetes as an index of population burden, particularly in least developed settings, with potential underestimation of true prevalence. However, with the exception of age, determinants of diagnosed and total diabetes were similar. We have demonstrated the feasibility of obtaining fasting blood samples from a high proportion of participants in our catchment area studies in Latin America, and aim to introduce these in future waves in other settings. The impact of diabetes upon older people, accounting for a substantial proportion of cases in most populations, has been relatively little studied. A better understanding may invigorate strategies to improve prevention and treatment. In an earlier analysis using the same 10/66 DRG baseline survey data sets, we reported a strong and consistent independent association between diagnosed diabetes and severe disability [14]. The median population attributable prevalence fraction was 4.1%, lower than that for dementia (25.1%), stroke (11.4%), limb weakness (10.5%), arthritis (9.9%), depression (8.3%), and eyesight problems (6.8%), but higher than that for chronic obstructive pulmonary disease (3.3%), hearing difficulties (2.2%) and ischaemic heart disease (0.8%). To capture the full impact of diabetes it will also be important, in future research, to assess the relative prevalence and incidence of diabetes complications, including renal, retinal and neuropathic pathology. Data on diabetes mortality among older people are particularly sparse. The decline in diabetes prevalence among the oldest old, also noted in other studies [3,5] is consistent with reduced survival, but might also be accounted for by lower incidence, or, for diagnosed diabetes, by underdetection. Widely-cited reports of substantial attributable mortality are based only on diabetes as an underlying cause on death certificates [15]. In the US national Second Longitudinal Study of Aging there was a modest statistically significant independent effect of diabetes on subsequent mortality among those aged 70 and over [16]. Catchment area survey designs such as that employed by the 10/66 DRG facilitate longitudinal research. With our incidence wave recently completed in most sites, we will report on associations between diabetes at baseline and incident dementia, stroke, dependence and mortality. First results, from Chennai, India, show no association between diagnosed diabetes and mortality [17].

## Conclusions

Our findings underline the need to boost detection in settings with a low prevalence of diagnosed diabetes, and to improve control of diagnosed diabetes everywhere. Strengthening primary care is key to this process [6]. For hypertension management, the benefits of family-based home health education from lay health workers, and annual training of general practitioners have recently been demonstrated in a cluster-randomised controlled trial in Pakistan [18]. For those with insulin-requiring diabetes there may be additional obstacles to be overcome, to ensure regular supplies of affordable insulin, needles and glucometers [19]. A low proportion of diagnosed diabetes cases received insulin in our rural and least developed sites. The well-documented 10/66 DRG catchment areas can serve as ‘population laboratories’ to monitor the course of the diabetes epidemic among older people in LMIC, and the effectiveness of efforts to improve prevention and control.

## Supporting Information

S1 Table Associations between sociodemographic, health and lifestyles factors and consenting to provide a blood sample, by site. (DOC)
