# Supplementary material for: The Prevalence, Correlates, Detection and Control of Diabetes among Older People in Low and Middle Income Countries. A 10/66 Dementia Research Group Population-Based Survey
Source: PLoS One. 2016 Feb 25;11(2):e0149616. doi: 10.1371/journal.pone.0149616 (PMC4767439; doi:10.1371/journal.pone.0149616)
Supplement: S1 Table — (DOC) [file pone.0149616.s001.doc]

Supplemental (online) table 1

Associations between sociodemographic, health and lifestyles factors and consenting to provide a blood sample, by site

|  | **Cuba** | **Dominican**  **Republic** | **Puerto**  **Rico** | **Venezuela** | **Mexico,**  **urban** | **Mexico,**  **rural** | **Peru,**  **urban** |
| --- | --- | --- | --- | --- | --- | --- | --- |
| Total sample | 2928 | 2010 | 2009 | 1965 | 1003 | 1000 | 1381 |
| Blood sample taken | 2355  (80.4%) | 1483  (73.8%) | 1569  (78.1%) | 1284  (65.3%) | 822  (82.0%) | 895  (89.5%) | 770  (55.8%) |
| Age (per 5 year band) | **0.98**  **(0.96-1.00)** | 0.99  (0.97-1.02) | **0.95**  **(0.93-0.97)** | 1.01  (0.98-1.04) | 0.98  (0.95-1.01) | 1.00  (0.98-1.02) | 0.96  (0.92-1.01) |
| Sex (male vs female) | **0.97**  **(0.93-1.00)** | **0.91**  **(0.86-0.96)** | 0.98  (0.94-1.03) | 0.97  (0.91-1.03) | 0.96  (0.90-1.01) | 0.99  (0.96-1.03) | 0.98  (0.89-1.07) |
| Education (per level) | **1.02**  **(1.01-1.04)** | 0.99  (0.96-1.02) | **1.03**  **(1.01-1.05)** | 0.99  (0.96-1.03) | **1.04**  **(1.01-1.07)** | 1.01  (0.98-1.03) | **1.08**  **(1.02-1.14)** |
| Assets (per quarter) | 1.02  (1.00-1.04) | 0.99  (0.96-1.02) | 1.03  (1.00-1.06) | 0.98  (0.94-1.01) | **1.05**  **(1.02-1.07)** | 1.01  (0.99-1.03) | **1.14**  **(1.08-1.20)** |
| Married | 1.01  (0.98-1.05) | 1.01  (0.95-1.08) | **1.06**  **(1.01-1.12)** | 1.02  (0.96-1.10) | 1.03  (0.97-1.10) | 1.01  (0.96-1.05) | **0.85**  **(0.78-0.94)** |
| Ever smoked | 0.98  (0.95-1.02) | 0.98  (0.93-1.03) | 0.97  (0.91-1.03) | 1.01  (0.94-1.07) | 0.95  (0.90-1.01) | 1.00  (0.96-1.05) | **1.20**  **(1.07-1.33)** |
| Arm circumference (per SD) | 1.01  (0.99-1.03) | 1.02  (0.99-1.04) | 1.02  (1.00-1.03) | 1.00  (0.96-1.04) | 0.98  (0.94-1.02) | 0.98  (0.95-1.01) | **0.77**  **(0.72-0.82)** |
| Obese  (meets metabolic syndrome criteria) | 1.01  (0.98-1.05) | **1.08**  **(1.02-1.13)** | 1.00  (0.97-1.04) | 1.03  (0.96-1.09) | 1.00  (0.94-1.06) | 0.97  (0.93-1.01) | 0.96  (0.87-1.06) |
| Self-reported diagnosed diabetes | 1.02  (0.98-1.07) | 1.01  (0.94-1.09) | 0.98  (0.94-1.03) | 0.96  (0.88-1.05) | 0.98  (0.91-1.04) | 1.01  (0.95-1.06) | 1.03  (0.87-1.21) |
| ICD-10 Depression | 1.00  (0.92-1.09) | **1.10**  **(1.03-1.17)** | 0.89  (0.74-1.08) | 0.94  (0.81-1.09) | 0.96  (0.82-1.12) | 1.02  (0.91-1.14) | **1.35**  **(1.17-1.55)** |
| Dementia | 1.00  (0.94-1.06) | 0.97  (0.90-1.06) | **0.72**  **(0.63-0.81)** | 0.94  (0.83-1.08) | 0.91  (0.81-1.03) | 0.97  (0.89-1.06) | 1.06  (0.89-1.25) |
| Stroke | 1.01  (0.95-1.08) | 101  (0.92-1.10) | **0.90**  **(0.81-0.99)** | 1.00  (0.88-1.13) | 0.92  (0.80-1.06) | **1.06**  **(1.00-1.13)** | **1.17**  **(1.01-1.36)** |
| Ischaemic heart disease | 0.96  (0.90-1.01) | 0.90  (0.75-1.07) | 0.92  (0.83-1.02) | 0.95  (0.82-1.09) | 0.90  (0.74-1.10) | 1.04  (0.91-1.20) | 0.92  (0.74-1.15) |
| Hypertension | 1.03  (0.99-1.08) | 1.03  (0.96-1.10) | **0.92**  **(0.88-0.96)** | 0.94  (0.87-1.01) | 0.97  (0.91-1.02) | 1.01  (0.96-1.05) | 1.07  (0.97-1.18) |
| Number of physical impairments | 1.01  (1.00-1.03) | **1.03**  **(1.01-1.04)** | 0.99  (0.97-1.00) | 1.00  (0.98-1.01) | **0.97**  **(0.95-1.00)** | 1.01  (1.00-1.02) | **1.13**  **(1.10-1.16)** |
| Severe disability  (above the 90^th^ centile for WHODAS 2.0) | 0.98  (0.92-1.05) | 0.98  (0.89-1.08) | **0.75**  **(0.66-0.84)** | 1.03  (0.93-1.14) | **0.80**  **(0.69-0.94)** | 1.00  (0.93-1.08) | 1.06  (0.91-1.24) |
